# Evaluation of Emerging *Fusarium* mycotoxins beauvericin, Enniatins, Fusaproliferin and Moniliformin in Domestic Rice in Iran

**Published:** 2015

**Authors:** Firouzeh Nazari, Michael Sulyok, Farzad Kobarfard, Hassan Yazdanpanah, Rudolf Krska

**Affiliations:** a *Food and Drug Organization, Iran University of Medical Sciences, Tehran, Iran. *; b *Center for Analytical Chemistry, Department for Agrobiotechnology (IFA-Tulln), University of Natural Resources and Life Sciences Vienna (BOKU), Konrad-Lorenz-Str. 20, A-3430 Tulln, Austria. *; c *Department of Medicinal Chemistry, School of Pharmacy, Phytochemistry Research Center, Shahid Beheshti University of Medical Sciences, Tehran, Iran. *; d *Department of Toxicology and Pharmacology, School of Pharmacy, Shahid Beheshti University of Medical Sciences, Tehran, Iran.*

**Keywords:** Beauvericin, Enniatins, Fusaproliferin, Moniliformin, Rice, Iran

## Abstract

The occurrence of emerging *Fusarium *mycotoxins beauvericin (BEA), enniatins (ENNs) (A, A1, B, B1), Fusaproliferin and moniliformin was evaluated by a liquid chromatography/electrospray ionization-tandem mass spectrometric (LC/ESI-MS/MS) technique in 65 domestic rice samples produced in Gilan and Mazandaran Provinces in Iran. The results showed that 46% of the samples were contaminated with at least one of the emerging mycotoxins. BEA was the most prevalent mycotoxin, which was found in 26 out of 65 rice samples at the concentrations up to 0.47 µg/Kg. Enniatin A1 which was the only member of ENNs was detected in the samples, occurred in 7.7% of samples with an average level of 0.06 μg/Kg. No detectable level of Fusaproliferin and moniliformin was found. This is the ﬁrst report concerning the contamination of Iranian domestic rice samples with the emerging *Fusarium* mycotoxins.

## Introduction

Mycotoxins are natural toxic secondary metabolites produced by various ﬁlamentous fungi *Aspergillus*, *Penicillium* and *Fusarium* species in a broad range of foodstuff (1). The existence of mycotoxins in food commodities is of major concern for public health because of their toxic properties including carcinogenicity, genotoxicity, immunotoxicity, mutagenicity and reproductive and developmental toxicity (2). *Fusarium* species produce the most common mycotoxins such as trichothecenes, fumonisins, moniliformin and zearalenone, as well as emerging mycotoxins like beauvericin (BEA) enniatins (ENNs), fusaproliferin (FUS) and moniliformin (MON) (3). There is only limited data available about these metabolites. This is not only due to their late recognition but especially because of the late understanding of their role as mycotoxins (4). Because of presence of high concentrations of emerging *Fusarium* mycotoxins in many food products, there is a great interest on these toxins. In addition, the European Food Safety Authority (EFSA) is collecting occurrence data for risk assessment at the moment.

**Figure 1:**
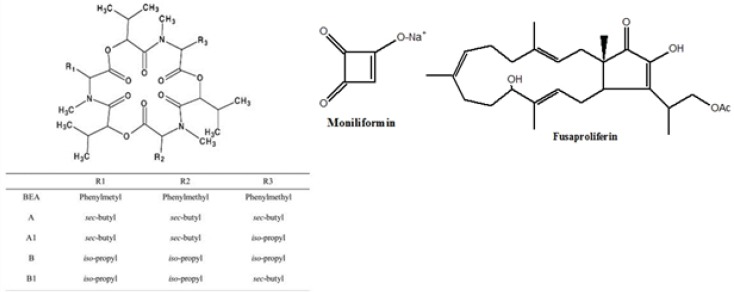
The chemical structures of beauvericin (BEA), enniatins (A, A1, B and B1), moniliformin and fusaproliferin.

BEA and ENNs are cyclic hexa depsipeptide mycotoxins (5) produced by different *Fusarium* species such as *F. avenaceum, F. subglutinans, F. oxysporum F. proliferatum, F. poae and F. tricinctum *(6, 7, 8)*. Fusaproliferinis* are produced by* F. antophilum, F. begoniae, F. bulbicola, F. circinatum, F. concentricum, F. succisae, *and* F. udum* (9). MON is sodium or potassium salt of 3-hydroxycyclobut-3-ene-1, 2-dione (10) and produced by several species of *Fusarium* including *F. avenaceum, F. tricinctum, *and *F. proliferatum* (11, 12). Chemical structures of BEA, enniatins, fusaproliferin and moniliformun have been shown in Figure 1.

All of them are bioactive compounds and elicit a wide range of different toxicological effects. BEA has shown genotoxic and cytotoxic effects in human lymphocytes and animal species, respectively (13, 14). It induces chromosomal aberrations, sister-chromatid exchanges and micronuclei formation (13). It also induces apoptosis (15, 16), mitochondrial dysfunction (17) and erythrocyte cell membrane scrambling (18). Finally, BEA has shown to be a speciﬁc cholesterol acyltransferase inhibitor (19). ENNs possess a wide range of biological activities. They have shown cytotoxic and apoptotic activity (20, 21). They are cationophoric compounds and specific inhibitors of acyl-coenzyme A cholesterol acyltransferase (ACAT) (22). The most well-known ENNs reported as natural contaminants are ENN A, A1, B and B1. Several other analogues (B2, B3, B4, D, E, F and G) have also been identiﬁed (23). ENNs have been found to have synergistic toxicity effects (15). ENN B is the most bioactive ENNs among B1, A and A1 and showed mitochondrial toxicity in isolated rat liver mitochondria (17).

FUS is a sesterterpene mycotoxin with a variety of toxic properties. This compound has been found to be toxic on Artemia salina (9, 24), human B lymphocytes (25), brine shrimp larvae and insect cells (26), and Caco-2 cells (27). It also has teratogenic and pathogenic effects (25).

MON is a potent inhibitor of the pyruvate dehydrogenase complex (28). MON induces acidosis and causes muscular weakness (29-30), cardiotoxicity (31), immunosuppression (32), and intestinal problems (33). 

The incidence of emergimg *Fusarium* mycotoxins has been reported in different foods such as maize (34, 35), wheat (36, 37), rice (38), grain-based products (39), cereal-based foodstuffs (40), breakfast, infant cereals (41), and barley (36). Although the majority of the studies are related to cereal and cereal products samples, there are some studies indicating the emergence of mycotoxin contamination in other food commodities. Abia *et al*. (2013) found BEA and ENNs in groundnuts, soybean, and groundnut soup in Cameroon (42). Sebastià *et al.* (2012) detected BEA and ENNs in tiger-nuts collected in Spain (43). Ezekiel *et al*. (2012) found BEA in peanut cake at concentrations up to 3.36 mg/Kg (44).

Rice (Oryza sativa L.) is one of the most important food crops worldwide especially for Iranian population. It can be ideal substrates for the growth of mycotoxin producing fungi during inappropriate storage conditions, moisture and insect infestation (45). 

Too little information is available regarding the incidence of BEA and EENS in rice samples (38, 46). Therefore, the aim of this study was to evaluate the presence of BEA, ENNs, MON and FUS in Iranian domestic rice using a highly sensitive and selective LC-MS/MS multi-mycotoxin method. 

## Experimental

*Reagents and equipment*

Methanol (MeOH) and acetonitrile (ACN) (both LC gradient grade) were purchased from Merck, Darmstardt, Germany and VWR, International Leuven, respectively; ammonium acetate (MS grade) and glacial acetic acid (p.a.) were obtained from Sigma Aldrich (Vienna, Austria). Water was puriﬁed successively by reverse osmosis and a Milli-Q plus system from Millipore (Molsheim, France).

Mycotoxin standards were dissolved in acetonitrile (ACN) predominantly to yield a multi-analyte working solution. This stock solution was subsequently used to prepare different working solutions for calibration. All standard solutions were stored at -20 C and were brought to room temperature before use. Extraction (ACN/water/glacial acetic acid 79:20:1, v/v/v) and dilution (ACN/ water/acetic acid 20:79:1, v/v/v) solvents were freshly prepared and kept at room temperature before use. Two eluents that contained 5 mM ammonium acetate each were prepared using MeOH/water/glacial acetic acid (10:89:1, v/v/v) (eluent A) and (97:2:1, v/v/v) (eluent B). 

*Sampling*

A total of 65 rice samples were collected randomly from local markets in Tehran, originally from Gilan Province and Mazandaran Province in northern Iran during April 2010 to April 2011. The minimum amount of samples was 1000 g. All samples were finely grounded and homogenized by milling (Romer Series II® Mill) and subsamples were stored at -20 ºC until analysis.

*Methodology*

Rice samples were analysed for the incidence and quantification of emerging *Fusarium* mycotoxins by LC-MS/MS according to Sulyok *et al.* (2006) (47). 

*Extraction*

Five grams of milled rice samples were extracted with 20 mL extraction solvent for 90 min at 180 rpm using a GFL 3017 rotary shaker (GFL 3017, Burgwedel, Germany). Aliquots of 500 µL extracts were transferred into glass vials containing an equal volume of the dilution solvent. After appropriate mixing, 5 µL of the diluted extracts were injected into the LC-MS/MS system.

*Chromatographic and mass spectrometric parameters*

Detection and quantiﬁcation were performed using a QTRAP 5500 LC-MS/MS System (AB SCIEX, Foster City, CA) equipped with Turbo Ion Spray electrospray ionization (ESI) source and a 1290 Series HPLC System (Agilent Technologies 1290 Inﬁnity).

 Chromatographic separation was performed at 25 °C on a Gemini^®^ C18 column, 150×4.6-mm *i.d*., 5-μm particle size, equipped with a C18 4×3-mm-i.d. security guard cartridge (all from Phenomenex, Torrance, CA, US). After an initial time of 2 min at 100% eluent A, the proportion of eluent B was increased linearly to 50% within 2-5 min and to 100% within 5-14 min, followed by a holding-time of 4 min at 100% eluent B and 2.5 min column re-equilibration at 100% eluent A pumped at a ﬂow rate of 1 mL/min.

The declustering potential (DP), entrance potential collision energy (CE) and collision cell exit potential (CXP) were optimized for each toxin (Table 1). ESI-MS/MS was performed in the scheduled multiple reaction monitoring (sMRM) mode both in positive and negative polarities in two separated chromatography runs per sample by scanning two fragmentation reaction per analyte with the following setting: source temperature 550  C; curtain gas 30 psi; ion source gas 1 (sheath gas) 80 psi, ion source gas 2 (drying gas) 80 psi, ion spray voltage - 4500 V, and +5500 V, respectively, collision gas (nitrogen) medium. The MRM detection windows were 54 and 96 s in the positive and negative ionization mode, respectively, and the cycle time was set to 1 s. 

Mycotoxins were quantified using external calibration (1/x weighted) and levels were later adjusted based on recoveries determined by spiking four samples. Limits of detection (LOD) and quantiﬁcation (LOQ) were estimated at the lowest concentration in spiked samples corresponding to a signal-to-noise ratio (S/N) of 3:1 and 10:1, respectively 10:1.

**Table 1:** Optimized ESI-MS/MS parameters

**Analyte**	**Precursor ion**	**Product ion**	**DP**	**EP**	**CE**	**CXP**	**Rt (min)**
Beauvericin	801.5[M+NH_4_]^+^ 806.5[M+Na]^+^	243.2 384.4	116 191	10 10	47 73	12 10	14.3
Enniatin A	699.4 [M+NH_4_]^+^	210.1/228	106	10	43/47	12/18	14.8
Enniatin A1	685 [M+NH_4_]^+^	210.1/228.2	96	10	41/49	8/20	14.5
Enniatin B	657.5 [M+NH_4_]^+^	196.3/214.1	81	10	45/47	18/18	14
Enniatin B1	671.4 [M+NH_4_]^+^	196/210	111	10	43/41	12/12	14.2
Fusaproliferin	445.2[M+H]^+^	368.1/350.1	96	10	25/29	10/10	13.3
Moniliformin	96.9 [M–H]−	41.2	-100	-10	-24	-5	3.2

DP: declustering potential, EP: enterence potential, CE: collision energy, collision CXP: cell exit potential, Rt: retention time

*Spiking experiment*

To determine the apparent recovery, spiking experiments were performed. In this regard, four blank (least contaminated) samples (each 0.25 g) were spiked with multi-analyte standard, thoroughly mixed and stored at room temperature in the dark cupboard about four hours to establish equilibration between the analytes and the matrix. Subsequently, one mL of extraction solvent was added and placed on a rotary shaker for 90 min at 180 rpm. After diluting 300 µL of extracts with an equal volume of the dilution solvent and mixing it for 30 s by vortex, 5 µL of the diluted extracts were injected into the LC-MS/MS system. 

## Results and Discussion

MS/MS parameters such as precursors and product ion, declustering potentials, collision energies and cell exit potentials were optimized for all analytes. All of the compounds exhibited precursor ions and product ions with reasonably high signal intensities in positive mode with the exception of MON which gave no signal in the positive ion mode. For each analyte, the polarity giving the most abundant product ions was selected; a second product ion was monitored for confirmation of the identity (with the exception of MON, which showed only one product ion). Table 1 summarizes the parameters of the optimized MRM transitions.

As shown in Table 1, among the seven emerging *Fusarium* mycotoxins, five mycotoxins (ENNB, ENNB1, ENNA1, ENNA and BEA) appeared as their ammonium adduct in mass spectra. Examined the chemical structures of these mycotoxins reveals that all these mycotoxins which appear as ammonium adduct, contain a 1,7,13-trioxa-4,10,16- triazacyclooctadecane ring as the backbone of their molecules. It could then be concluded that the presence of 18 membered ring of trioxa triazacyclooctadecane is responsible for the formation of ammonium adducts in mass spectra.

The recoveries of all mycotoxins ranged from 94 % to 111% (Table 2). It was observed that correlation coefficients (r2) were >0.99 for all the analyets.

**Table 2:** Method Performance Characteristics for Beauvericin, and Enniatins, moniliformin and fusaproliferinin rice samples.

Analyte	LOD^a^(µg/Kg )	LOQ^b^(µg/Kg )	Recovery (%)^c^
Beauvericin	0.02	0.06	101.9 ± 5.1
Enniatin A	0.02	0.06	98.1 ± 3.8
Enniatin A1	0.02	0.06	94.1 ± 2.6
Enniatin B	0.02	0.06	101.8 ± 5.1
Enniatin B1	0.02	0.06	100.5 ±3.9
Moniliformin	0.08	0.25	105.3 ± 2.1
Fusaproliferin	0.02	0.06	110.8 ± 23.1

a LOD, (S/N = 3:1) expressed as μg/Kg sample

b LOQ (S/N = 10:1) expressed as μg/Kg sample

c Mean and standard deviation of four samples

*Occurrence of emerging Fusarium mycotoxins in rice samples*

Despite the great concern about the toxicity of these toxins and their natural incidence in different food stuff, no maximum limits and tolerable daily intakes (EDI) have been established for them.

In this study, the occurrence of BEA, ENNs, MON and FUS was evaluated in rice samples and the results have been shown in Table 3. The results indicated that 46% of samples were contaminated with at least one of the emerging mycotoxins.

**Table 3:** Occurrence of emerging *Fusarium* mycotoxins in Iranian rice samples

Analyte	Incidence (%)	Mean(μg/Kg)		Max(μg/Kg)		Daily intake(ng/Kgbw/day)	
						Mean	Max
Beauvericin	26 (40)	0.11	0.47		0.2		0.86
Enniatin A1	5 (7.7%)	0.06	0.08		0.11		0.15
Enniatin A	nd	-	-		-		-
Enniatin B	nd	-	-		-		-
Enniatin B1	nd	-	-		-		-
Moniliformin	nd	-	-		-		-
Fusaproliferin	nd	-	-		-		-

nd: not detected

*Beauvericin*

The results showed that the most prevalent mycotoxin was BEA, which was found in 26 out of 65 rice samples at the concentrations up to 0.47 µg/Kg.

There are limited data available on the occurrence of emerging mycotoxins in rice. Meca *et al*. (2010) found BEA contamination in rice samples from Spain at a level of 11.78 mg/Kg (46). In Morocco, Sifou *et al*. (2011) found that BEA was present in 75.7% of rice samples, and contamination level up to 26.3 mg/Kg was observed (38)**. **Nazari *et al.* found that 88% of imported rice samples to Iran were contaminated with low level of BEA (range: 0.01 to 1.56 µg/Kg) (unpublished data). Natural occurrence of BEA in other food commodities has been reported in different countries. Logrieco *et al*. (2002) reported that BEA was found in all 13 wheat samples from Finland in the concentration range of 0.64 to 3.5 µg/g (6). In another study in Finland, BEA was detected in 95% of 38 grain samples (39). Mahnine *et al.* (2011) reported the presence of BEA in 5.8% of 68 cereal derivatives products (breakfast cereals and infant cereals) from Morocco with a maximum level of 10.6 mg/Kg (41).

*Enniatins*

In our study, ENNA1 was the only ENN among ENNB, ENNB1, ENNA1, and EENA, which was found in the samples. ENNA1 contamination occurred in 7.7% of rice samples with an average level of 0.06 μg/Kg. Sifou *et al*. (2011) reported that total ENNs was found in 50% of rice samples (38). ENNB was the most prevalent ENN which found in 30% of the samples, while ENNB1, ENNA and ENNA1 were found in 24.6% (ranged: 4.4 to 26.2 mg/Kg), 22.8% (ranged: 8.4 to 119.5 mg/Kg) and 5.7% (ranged: 56.2 to 448.7 mg/Kg) of total samples, respectively (38). Nazari *et al*. reported that ENNs (ENNB, ENNB1, ENNB2, and ENNA1) were detected in 9% of imported rice samples to Iran with contamination frequencies of 3%, 3%, 1%, and 2% for ENNB, ENNB1, ENNB2, and ENNA1, respectively (unpublished data). Meca *et al*. (2010) detected the occurrence of ENA1 (814.42 mg/Kg) and ENB (7.95 mg/Kg) in one sample of rice from Spain (46).

*Fusaproliferin*

As for FUS, no detectable amount of FUS was identified in the Iranian rice samples. The low incidence of FUS in rice has been previously reported by others (38, 41, and 46). In Morocco, 4.3% of rice samples were contaminated with FUS at the concentration range of 0.2 to 19.6 mg/Kg (38). In another study in Morocco, Mahnine *et al*. (2011) reported that one rice-based infant cereal was contaminated to FUS at the concentration level of 7.4 mg/Kg (41). In Spain, Meca *et al*. (2010) reported that FUS was found in one rice sample at the level of 3.17 mg/Kg (46). 

*Moniliformin*

In our study, no detectable level of MON was found in samples. Data on the occurrence of MON in rice is limited. Nazari *et al.* (unpublished data) reported that only one out of 100 imported rice samples was contaminated to MON at the level of 8.25 µg/Kg. Yu *et al.* (1995) analyzed 123 rice samples and found MON was present in 8 samples ranging from 73.6 to 265.3 µg/Kg (48).

*Estimated daily intake of emerging fusarium mycotoxins*

In Iran, two studies regarding estimation of daily intake of AFB1 and DON in foods have been done (49-50). The results obtained in the present study were used to estimate the daily intake of emerging fusarium mycotoxins from rice by the Iranian population. Exposure to mycotoxins for each type of food depends on the mycotoxins level in food and the amount of food consumed. In this study, the consumption rate of rice (110 g per day per person) was based on a consumption survey performed in Iran during 2001-2003 (51) and body weight (b.w) for adults is assumed 60 Kg. 

In this study, the EDI of BEA and ENN A1 were 0.2 and 0.11 ng/Kg b.w/day, respectively, and maximum daily intake of BEA and ENN A1 were 0.8 and 0.15 ng/Kg b.w/day, respectively.

Since JECFA has not set a provisional maximum tolerable daily intake (PMTDI), it is not possible to conﬁrm that these contamination levels of ENs and BEA represent a risk for Iranian consumers.

## Conclusion

For the ﬁrst time, the presence of emerging *Fusarium* mycotoxins especially BEA in Iranian rice was reported. Although the contamination level of BEA was low, considering the toxic effects of BEA, high incidence of the toxin, and high consumption of rice in Iran, BEA may pose health effects to Iranian population. The results of present study indicate the potential health threat due to the contamination of rice with some non-classic mycotoxins which might be neglected in conventional mycotoxin monitoring. Therefore, in Iran, more comprehensive mycotoxin monitoring programs may be needed in rice and other food commodities, in which emerging* Fusarium* mycotoxins, particularly BEA, should be included. In addition, the effect of different methods of food processing on the level of these new emerging mycotoxins in foods**, **and environmental conditions which inﬂuence the occurrence of them should be studied.
